# Insights into the Utility of the Focal Adhesion Scaffolding Proteins in the Anaerobic Fungus *Orpinomyces* sp. C1A

**DOI:** 10.1371/journal.pone.0163553

**Published:** 2016-09-29

**Authors:** Shelby Calkins, Noha H. Youssef

**Affiliations:** Department of Microbiology and Molecular Genetics, Oklahoma State University, Stillwater, OK 74078, United States of America; Istituto per la Ricerca e la Cura del Cancro di Candiolo, ITALY

## Abstract

Focal adhesions (FAs) are large eukaryotic multiprotein complexes that are present in all metazoan cells and function as stable sites of tight adhesion between the extracellular matrix (ECM) and the cell’s cytoskeleton. FAs consist of anchor membrane protein (integrins), scaffolding proteins (e.g. α-actinin, talin, paxillin, and vinculin), signaling proteins of the IPP complex (e.g. integrin-linked kinase, α-parvin, and PINCH), and signaling kinases (e.g. focal adhesion kinase (FAK) and Src kinase). While genes encoding complete focal adhesion machineries are present in genomes of all multicellular Metazoa; incomplete machineries were identified in the genomes of multiple non-metazoan unicellular Holozoa, basal fungal lineages, and amoebozoan representatives. Since a complete FA machinery is required for functioning, the putative role, if any, of these incomplete FA machineries is currently unclear. We sought to examine the expression patterns of FA-associated genes in the anaerobic basal fungal isolate *Orpinomyces* sp. strain C1A under different growth conditions and at different developmental stages. Strain C1A lacks clear homologues of integrin, and the two signaling kinases FAK and Src, but encodes for all scaffolding proteins, and the IPP complex proteins. We developed a protocol for synchronizing growth of C1A cultures, allowing for the collection and mRNA extraction from flagellated spores, encysted germinating spores, active zoosporangia, and late inactive sporangia of strain C1A. We demonstrate that the genes encoding the FA scaffolding proteins α-actinin, talin, paxillin, and vinculin are indeed transcribed under all growth conditions, and at all developmental stages of growth. Further, analysis of the observed transcriptional patterns suggests the putative involvement of these components in alternative non-adhesion-specific functions, such as hyphal tip growth during germination and flagellar assembly during zoosporogenesis. Based on these results, we propose putative alternative functions for such proteins in the anaerobic gut fungi. Our results highlight the presumed diverse functionalities of FA scaffolding proteins in basal fungi.

## Introduction

In eukaryotes, focal adhesions are sites of stable contacts with the ECM and subsequent polymerization of the cell’s cytoskeleton. They mediate interaction between the ECM and the cell interior by promoting cell anchorage and mechanical adhesion to the ECM, as well as act as signaling milieu where signaling proteins are concentrated at sites of integrin binding and connect the cell’s cytoskeleton to the ECM. FAs are comprised of large multiprotein complexes that are mediated by integrins, heterodimeric membrane proteins that act as the point of matrix-cytoskeleton connection [1]. The structure of the integrin adhesome and the mechanism of the focal adhesion process have been extensively studied in metazoan cell culture lines [1–3]. The process is mediated by a complex set of proteins. For the sake of simplicity, we highlight the major proteins mediating the process. For a more detailed view, the reader is referred to [4]. Briefly, the process is initiated in the presence of an ECM protein ligand, e.g. fibronectin that binds to the ECM receptor integrin. This integrin-ECM bond recruits the scaffolding protein talin to the focal adhesion site, which in turn binds actin microfilaments and functions to strengthen the integrin-ECM bond. Integrin-talin-actin complexes recruit additional components such as focal adhesion kinase (FAK), paxillin, and Src-family kinases (SFKs) to integrin tails thereby revealing binding sites for other proteins, such as vinculin. The integrin-cytoskeleton link is further stabilized by the recruitment of the IPP complex, comprising integrin-linked kinase (ILK), parvin, and PINCH, to promote cytoskeleton linkage and integrin signaling. Actin crosslinking occurs via α-actinin, which orchestrates the elongation and growth of focal adhesions.

**Fig 1:**
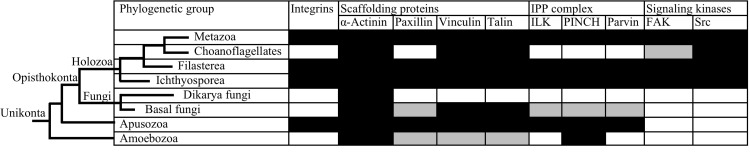
Genomic evidence for focal adhesion complex components in metazoan and non-metazoan Unikonts. Results shown are from [8–10, 30], and are based on the genomic analysis of several Metazoa representatives, the Choanoflagellates representatives *Monosiga brevicollis* and *Proterospongia* sp., the Filasterea representative *Capsaspora owczarzaki*, the Ichthyosporea representative *Sphaeroforma*, several representatives of the Dikarya fungi, the basal fungi representatives *Allomyces macrogynus* (Blastocladiomycota), *Spizellomyces punctatus*, *and Batrachochytrium dendrobatidis* (Chytridiomycota), *Orpinomyces* sp. C1A, *Anaeromyces robustus*, *Neocallimastix californiae*, and *Piromyces finnis* (Neocallimastigomycota), the Apusozoa representative *Amastigomonas* sp., and several representatives of the Ameobozoa. The dendogram is not drawn to scale and only serves to show the relationships between the different groups. Cells shaded in black denote clear homologues were identified in all representative genomes, cells shaded in grey denote clear homologues were identified in some but not all representative genomes, and cells shaded in white denote no homologues were identified in any of the representative genomes.

**Fig 2:**
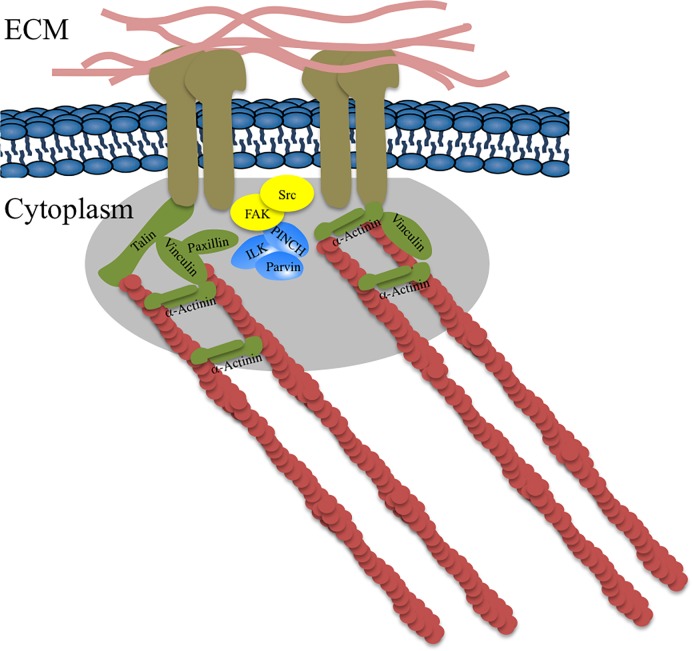
A simplified schematic of the focal adhesion machinery in Metazoa. Focal adhesion proteins are color coded as follows: Integrins (ECM receptors) are brown, Scaffolding proteins are green, proteins of the IPP complex are blue, signaling kinases are yellow. F-actin polymers are shown in red and proteins of the extracellular matrix are shown in pink.

Focal adhesion is essential for multicellularity since it enables cells to attach to components of the ECM [5]. Accordingly, it was thought until recently that the integrin adhesome and its function in focal adhesion was metazoan specific [6, 7]. However, this view was challenged when homologues of FA proteins were identified in the genomes of several unicellular non-metazoan Holozoa; such as the Choanoflagellates *Proterospongia* sp. and *Monosiga brevicollis*, the Filasterea *Capsaspora owczarzaki*, the Ichthyosporea *Sphaeroforma*, the genome of the Apusozoa (the Opisthokonta sister group) *Amastigomonas* sp., and genomes of several representatives of the Amoebozoa, (Fig 1, and [8, 9]). Further, in Fungi, the Holozoa sister group within the Opisthokonta, homologues of FA proteins were also identified in the genomes of various basal fungal phyla, but not the Dikarya (Ascomycota and Basidiomycota). Interestingly while the pattern of occurrence of FA components varies between different basal fungal lineages (Fig 1), all of them invariably lack homologues for integrin and the signaling kinases FAK and Src, but encode for scaffolding proteins. In the absence of integrin and the signaling kinases, the connection between the cytoskeleton and the ECM is lost and hence the known function of focal adhesions might not be realized (Fig 2). Therefore, it is currently unclear whether the focal adhesion components in basal fungi are nonfunctional and only represent a remnant of a once complete FA machinery, mediate adhesion with the help of a yet-unidentified integrin functional homologue, or are involved in some hitherto unrecognized function as part of a non-adhesion related machinery.

The anaerobic gut fungal isolate *Orpinomyces* sp. strain C1A encodes a partial FA machinery [10]. The genome contains homologues of the IPP complex and the four scaffolding proteins talin, vinculin, paxillin, and α-actinin, but lacks homologues for integrin, FAK, and Src. Here, we reasoned that transcriptional patterns of genes encoding this partial FA machinery in strain C1A under various growth conditions and developmental stages could provide preliminary insights into the functionality and putative biological role(s) of these proteins. We show for the first time in basal fungi that the genes encoding scaffolding proteins are indeed transcribed during growth in the absence of the upstream integrin and the signaling kinases. Phylogenetic analyses, conservation of functional domains, and comparative modeling all suggest that the predicted proteins would be functional. Based on transcriptional levels at different stages of the life cycle of this fungus, we discuss the possible cellular roles of scaffolding proteins in C1A.

## Materials and Methods

### Organism and culture media

Strain C1A was originally isolated from an Angus steer [10]. Cultures of C1A were regularly transferred twice a week for maintenance in rumen fluid media with cellobiose as the carbon source [11]. Where indicated, cellobiose was replaced with microcrystalline cellulose as the C source. Prior to inoculation, the media were amended with an anaerobic antibiotic mixture of kanamycin, penicillin, streptomycin, and chloramphenicol with final concentrations of 50 μg/mL, 50 μg/mL, 20 μg/mL, and 50 μg/mL, respectively.

### FA components in C1A genomes, phylogenetic analysis, and functional domain prediction

We queried the genome of strain C1A [10, 12] to identify FA-related genes/transcripts. Phylogenetic analysis of the predicted scaffolding proteins was conducted to identify their closest relative and examine whether their topologies are in agreement with the organismal phylogeny. Individual multiple sequence alignments (MSA) were constructed for each of the predicted proteins in Mega [13]. When possible, beside metazoan homologues, predicted proteins from non-metazoan eukaryotic representatives (including basal fungi) were added to the alignment. The resulting MSA was utilized to construct maximum likelihood trees using the best substitution model identified using the calculated values for the Akaike information criterion (AIC), Bayesian information criterion (BIC), and likelihood ratio as tested in Mega [13].

All predicted proteins were checked for functional domain structure and organization by querying against the Pfam database [14]. We used Phyre2 [15] homology modeling to construct pairwise sequence alignments with secondary and tertiary structure predictions for α-actinin, talin, and vinculin. The predicted tertiary structure models were visualized in PyMol [16], and each of the predicted models was superimposed to the corresponding template structure used for structure predictions. The protein data bank [17] (PDB) ID’s for the templates utilized were as follows: α-actinin (PDB ID: 1SJJ, origin: chicken gizzard smooth muscle), talin (PDB ID’s: 1SJ7, 2L10, 2JSW, 2KVP, 2QDQ, origin: *Mus musculus*), and vinculin (PDB ID: 1TR2, origin: *Homo sapiens*). When the predicted paxillin sequence was searched against the PDB database, no hits with structural data were identified. Accordingly, for paxillin a secondary and tertiary structure prediction was not possible.

### Transcriptional studies of genes encoding scaffolding proteins in C1A

In addition to the mere documentation of transcription of genes encoding scaffolding proteins, we sought to use real time PCR to examine and analyze the transcriptional levels of such genes under various growth conditions and various developmental stages. Our aim was to examine whether the transcriptional patterns observed support a putative role for FA scaffolding proteins in an adhesion-related processes, or whether they would be involved in developmental-stage specific function other than adhesion, e.g. in flagellar assembly, as previously suggested for the ciliated cells of *Xenopus tropicalis* [18], or hyphal tip growth as previously speculated in the basal fungus *Allomyces arbuscula* [19].

### Transcriptional patterns in presence and absence of an ECM trigger for adhesion

To examine the putative involvement of the scaffolding proteins in adhesion-related processes, we compared the transcription levels of their genes in presence and absence of an extracellular matrix trigger for FA. Fungi differ from animal cells in that they lack a well-defined protein-rich extracellular matrix but possess a polysaccharide-rich cell wall [20]. However, in zoosporic fungi (e.g. members of Chytridiomycota, Neocallimastigomycota, Blastocladiomycota, and Monoblepharidomycota), the zoospore stage was shown to have some type of polysaccharide-rich [21] extracellular matrix in the form of a cell coat covering the zoospore body and excluding the flagellar axoneme [22]. Therefore, in contrast to in-vitro studies of focal adhesions in Metazoa that used a protein, usually fibronectin, as the extracellular trigger for focal adhesion [23], we opted for using a polysaccharide (microcrystalline cellulose; MCC) as the extracellular trigger for FA in C1A. The choice of MCC was based on the macroscopic and microscopic observations of C1A hyphal tight attachment to MCC during growth. The hypothesis here is that if the scaffolding proteins are implicated for adhesion, then their genes are expected to be differentially up-regulated when C1A is grown in the presence of the extracellular polysaccharide MCC as opposed to a soluble substrate such as cellobiose. To this end, C1A cultures were grown in rumen fluid media with cellobiose, as opposed to the insoluble MCC as the carbon source. Cultures of C1A on cellobiose and MCC were sacrificed at mid log phase (51 hours post inoculation, based on biomass determination in preliminary experiments), and the biomass was used for total RNA extraction and for studying the transcriptional levels of the FA scaffolding genes as detailed below.

### Transcriptional patterns of scaffolding genes at various developmental stages

As described above, an alternative hypothesis posits that scaffolding proteins might be involved in a non-adhesion-specific process during the life cycle of strain C1A, as shown before in ciliated cells of the metazoan *Xenopus tropicalis* [18], or as previously suggested for the basal fungus *Allomyces arbuscula* [19]. To examine this hypothesis, we quantified the transcriptional levels of talin, α-actinin, vinculin, and paxillin, at four distinct developmental stages in C1A: flagellated spores, encysted non-flagellated germinating spores, active sporangia during zoosporogenesis, and late inactive sporangia samples.

**Fig 3:**
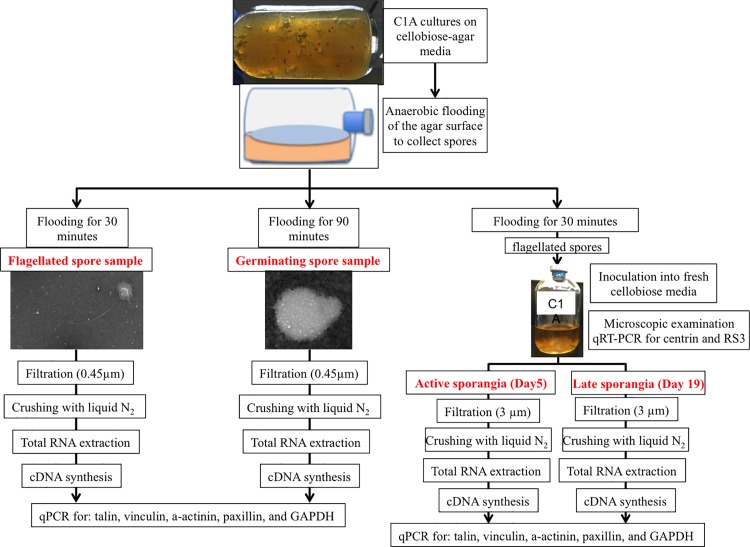
Schematic of the protocol used to collect the different developmental stages of C1A employed for the transcriptional study.

While separation and collection of life cycle stages is a routine practice in aerobic zoosporic fungi, and involves zoospore collection and growth synchronization [24, 25], we had repeated difficulties replicating such synchronization starting from flagellated zoospores in the anaerobic fungal representative strain C1A due to the observed wide range of zoospores encystment and germination time following introduction to fresh media. Therefore, we developed alternative approaches for the collection of various developmental stages in strain C1A. The procedure (Fig 3) was partly described in [11], and involves growing C1A cultures on cellobiose rumen fluid media in presence of 2% agar followed by flooding the agar surface with sterile anoxic water (SAW) to promote spore release from the sporangia. Variation in time between culture flooding and collection of the released spores was used to obtain either 100% swimming spores-only sample (flooding incubation time of 30 minutes), or a >90% non-flagellated germinating spore sample (flooding incubation time of 90 minutes).

To obtain samples representative of active and late sporangia, we started from the swimming spores collected as described above (flooding agar surface followed by 30 minutes incubation). The obtained spores were used to inoculate fresh rumen fluid cellobiose media, and growth was monitored microscopically (using the phase contrast lens of an Olympus BX51 microscope after staining with lactophenol-cotton blue stain solution) on a daily basis for 19 days. Judging by cues from the microscopic examination we sacrificed samples on days 1, 2, 5, 15, and 19 following inoculation as those days coincided with various stages of development as shown in Table A in S1 File. Further, it has been shown before [26] that the whole basal body carrying the flagella is shed along with the axoneme during encystment of flagellated spores. The basal body and the axoneme are then re-built during zoosporogenesis in active sporangia. Accordingly, we hypothesized that the transcriptional levels of genes encoding axoneme-specific, as well as basal body-specific proteins should correspond to the level of zoosporogenesis. Therefore, in conjunction with the microscopic examination, we followed the transcriptional levels of RS3 (encoding an axoneme-specific protein [27]), and centrin (encoding basal body and nuclear cap-specific protein [28]) in the samples collected above (days 1-2-5-15-19). Samples with the highest transcriptional levels of RS3 and centrin genes and microscopic evidence of active zoosporogenesis were used as representatives of active sporangia, while late samples that showed no microscopic evidence of zoosporogenesis as well as very low to no transcription of RS3 and centrin were used as representatives of late sporangia samples.

### RNA extraction, cDNA synthesis, and quantitative RT-PCR

Collected samples were vacuum filtered using sterile 3 μm (for biomass and sporangia samples) or 0.45 μm (for spore-only samples) filters and the obtained biomass was lysed by crushing with a sterile mortar and pestle upon submersion in liquid nitrogen. Crushed cells were used for total RNA extraction using MasterPure^TM^ Yeast RNA Purification Kit (Epicentre®, Madison, WI) according to manufacturer’s instructions. Total RNA reverse transcription (cDNA synthesis) was performed using the Superscript III First-Strand Synthesis System for RT-PCR (Invitrogen^TM^, Carlsbad, CA) with Oligo(dT)_20_ according to manufacturer’s instructions. Genes’ transcriptional levels were investigated using quantitative RT-PCR using a MyIQ thermocycler (Bio-Rad Laboratories, Hercules, CA) and SybrGreenER® qPCR mix (Invitrogen^TM^, Carlsbad, CA). Primers targeting α-actinin, talin, vinculin, paxillin, RS3, and centrin cDNA were designed using the OligoPerfect^TM^ Designer tool (Invitrogen^TM^, Carlsbad, CA) and their specificity was tested *in-silico* using the standalone NCBI Blastn [29] against all coding sequences of C1A. Primer sequences, their target accession number, as well as amplified regions are shown in Table 1. The reactions contained 1μl of C1A cDNA, and 0.5 μM each of the forward and reverse primers. Reactions were heated at 50°C for 2 min, followed by heating at 95°C for 8.5 min. This was followed by 70 cycles, with one cycle consisting of 15 s at 95°C, 60 s at 50°C, and 30 s at 72°C. Using the ΔCt method, the number of copies of each gene is reported relative to the number of copies of the housekeeping gene glyceraldehyde-3-phosphate dehydrogenase (GAPDH) used as the normalizing control.

**Table 1:** Quantitative PCR primers used for cDNA amplification.

Transcript/ protein	Accession number	Forward primer sequence	Reverse primer sequence	Amplified region	Function	Use in this study
Talin	KX463728	GCCGCATCTAAAAACGTAGC	CGGTGAAAGCATCGGTATCT	6100–6287 (188 bp)	Scaffolding protein	Transcriptional study
Vinculin	KX463730	CCGCCGAAAAGAATGATTTA	TTCAATGACTGGTGGCTTTG	29–162 (134 bp)	Scaffolding protein	
Paxillin	KX463729	TCCCAGCTGGTAGTGGTTTC	TTCAGTTGGGAAGGAGCAAC	569–759 (191 bp)	Scaffolding protein	
α-actinin	KX463732	CACAATTTGCTGCCTTTGAA	CCTTCTGGTGGGTTGTATGG	515–658 (144 bp)	Scaffolding protein	
GAPDH	KX463731	ATTCCACTCACGGACGTTTC	CTTCTTGGCACCACCCTTTA	134–339 (206 bp)	Housekeeping gene	Normalizing control for qPCR
RS3	KX463733	TTTTTGCCATGGCTTACTCA	TGGTTTTCCTTCATCGCATT	262–489 (228 bp)	Axoneme component	Zoosporogenesis monitoring
Centrin	KX463734	TTCACAGACAATCACGTTCAAA	TCAGCCATTTTTGCTGACAT	14–285 (272 bp)	Basal body component	

## Results

### Phylogeny, Pfam domain analysis, and structure modeling of FA proteins in C1A

**Fig 4:**
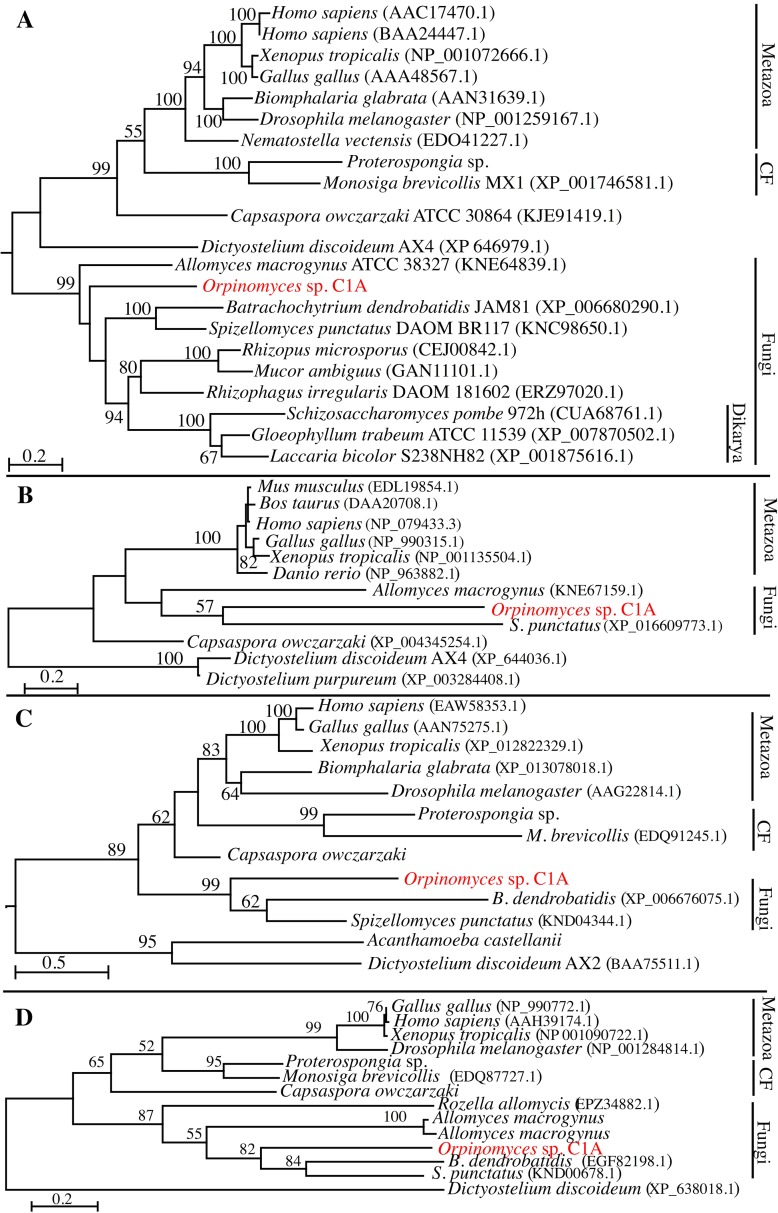
Maximum likelihood phylogenetic analysis of C1A predicted scaffolding proteins. All evolutionary analyses and model selections were conducted in MEGA7 [13]. Trees are drawn to scale, with branch lengths measured in the number of substitutions per site. Bootstrap values, in percent, are based on 100 replicates and are shown for branches with >50% bootstrap support. Trees are shown for: (A) α-actinin based on the JTT model with a discrete Gamma distribution (variable site γ shape parameter = 2.0327). Analysis involved 21 amino acid sequences, with a total of 535 positions in the final dataset. (B) Paxillin based on the Dayhoff model with a discrete Gamma distribution (variable site γ shape parameter = 1.7755). Analysis involved 12 amino acid sequences, with a total of 535 positions in the final dataset. (C) Talin based on the Le_Gascuel_2008 model with a discrete Gamma distribution (variable site γ shape parameter = 3.0802). Analysis involved 13 amino acid sequences, with a total of 441 positions in the final dataset. (D) Vinculin based on the Le_Gascuel_2008 model with a discrete Gamma distribution (variable site γ shape parameter = 3.4035). Analysis involved 14 amino acid sequences, with a total of 145 positions in the final dataset.

All four predicted scaffolding proteins from C1A (α-actinin, talin, vinculin, and paxillin), showed a topology consistent with C1A organismal phylogeny. In all maximum likelihood trees, C1A proteins formed a well-supported cluster with proteins from other basal fungi (and Dikarya fungi in case of α-actinin) (Fig 4). Likewise, all Metazoan proteins clustered together with strong bootstrap support, with the Choanoflagellates (*Monosiga* and *Proterospongia*) and the Filasterea (*Capsaspora*) proteins forming sister groups. However, the position of proteins from Amoebozoan origin was not consistent across trees (Fig 4). When other Neocallimastigomycota genomes/transcriptomes [30] were queried for FA components homologues, a pattern similar to that of C1A was detected, where genes encoding the IPP complex components as well as the scaffolding proteins were identified (Table B in S1 File) with no homologues for integrin, FAK, or Src.

**Fig 5:**
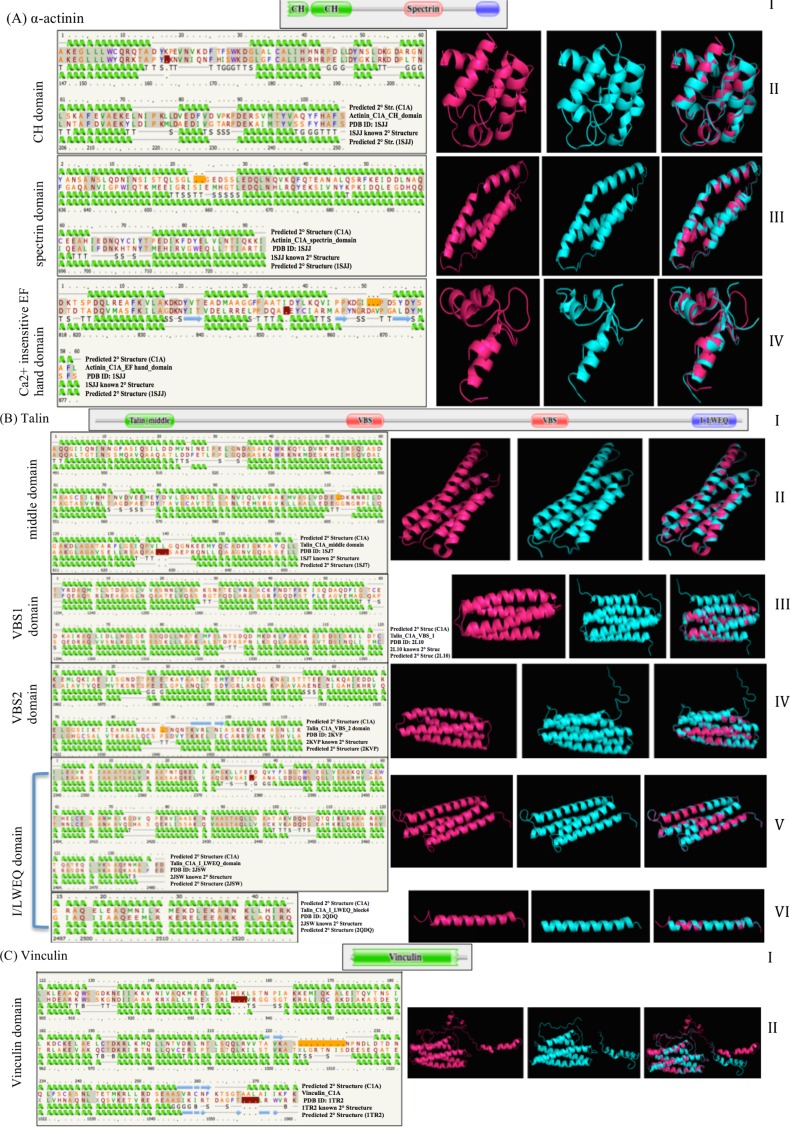
C1A predicted scaffolding proteins functional domain structure and organization, and predicted protein structure modeling. Results are shown for (A) α-actinin, (B) talin, and (C) vinculin. For each predicted protein, the first row (I) corresponds to the predicted Pfam domain organization. This is followed by 1–5 rows (II-VI) each corresponding to a functional domain. On the left of each of these rows, secondary structure alignments of C1A predicted domian compared to a template’s known and predicted secondary structure are shown. On the right of each row, predicted tertiary structures of C1A domains are shown in pink, compared to the template’s known tertiary structure in cyan. Superimposed structures are also shown. Row (A-II): predicted C1A α-actinin CH domain structure compared to PDB: 1SJJ from *Gallus gallu*s. Row (A-III): predicted C1A α-actinin spectrin domain structure compared to PDB: 1SJJ from *Gallus gallu*s. Row (A-IV): predicted C1A α-actinin Ca^2+^ insensitive EF hand domain structure compared to PDB: 1SJJ from *Gallus gallu*s. Row (B-II): predicted C1A talin middle domain structure compared to PDB: 1SJ7 from *Mus musculus*. Row (B-III): predicted C1A talin VBS1 domain structure compared to PDB: 2L10 from *Mus musculus*. Row (B-IV): predicted C1A talin VBS2 domain structure compared to PDB: 2KVP from *Mus musculus*. Row (B-V): predicted C1A talin I/LWEQ domain (Blocks 1–3) structure compared to PDB: 2JSW from *Mus musculus*. Row (B-VI): predicted C1A talin I/LWEQ domain (Block 4 comprizing the dimerization domain) structure compared to PDB: 2QDQ from *Mus musculus*. Row (C-II): predicted C1A vinculin domain structure compared to PDB: 1TR2 from *Homo sapiens*.

The results of Pfam domain analysis are shown in Table 2 and Fig 5 and are detailed in S1 File. Analysis identified domain organizations that are consistent with functional proteins from Metazoan origin. This includes 2 Calponin homology (CH) (Pfam 00307) domains, one spectrin repeat (Pfam 00435), and one Ca^2+^ insensitive EF hand (EF) domain (Pfam08726) for C1A predicted alpha-actinin [31], a talin middle domain (Pfam 09141), 2 vinculin binding site domains (Pfam 08913), and an I/LWEQ domain (Pfam 01608) for C1A predicted talin, and vinculin family domain (Pfam 01044) for C1A predicted vinculin (Fig 5). C1A predicted paxillin available sequence was 5’ partial and did not span the paxillin domain itself, but comparison against the Pfam database identified 4 LIM domains (Pfam 00412) consistent with Metazoan paxillin C-terminal region [32] (Fig A in S1 File). Within the recognized domains, several characteristic residues that were shown before to be conserved and essential for activity [32–34] were identified (Table 2).

**Table 2:** Results of C1A scaffolding proteins comparison to the Pfam database, as well as secondary and tertiary structure predictions.

C1A protein (accession No.)	Blastp First hit Accession No. (organism)	Pfam comparison			Secondary structure prediction and comparative modeling					Reference
		Pfam domain	Residues in C1A protein	Residues important for activity	Template PDB ID (organism)	% similarity	% modeling confidence	Predicted 3° structure	RMSD of super-imposition	
α-actinin (KX463732)	KXS11038 (*Gonopodya prolifera*)	CH (Pfam 00307)	1–55		Partial, not modeled					
		CH (Pfam 00307)	63–171		1SJJ (*Gallus gallus*)	60%	100%	4 alpha helices	0.941	[39]
		Spectrin repeat (Pfam 00435)	312–413	Aromatic residues Y317 and Y397, and L428	1SJJ (*Gallus gallus*)	27%	98.93%	triple-helical coiled-coil motif	0.956	[41]
		Ca^2+^ insensitive EF hand (Pfam 08726)	505–566		1SJJ (*Gallus gallus*)	32%	99.6%	helix-loop-helix motif	1.119	[35]
Talin (KX463728)	XP_016612383 (*Spizellomyces punctatus*)	middle domain (Pfam 09141)	100–262		1SJ7 (*Mus musculus*)	30%	100%	bundle comprised of 5 alpha helices	0.116	[40]
		vinculin binding site (Pfam 08913)	838–961		2L10 (*Mus musculus*)	30%	100%	4 alpha helices	0.902	[38]
		vinculin binding site (Pfam 08913)	1455–1578		2KVP (*Mus musculus*)	21%	97%	4 alpha helices	0.653	[38]
		I/LWEQ domain (Pfam 01608)	1952–2139. Block 1: 1952–1977, Block 2: 1996–2018, Block 3: 2029–2053, Block 4: 2109–2128	4-block structure: • Block 1: several conserved branched chain residues, Q1975 • Block 2: W1996 and several non-polar residues • Block 3: E2029, Q2044, K2052.	2JSW (*Mus musculus*)	53%	100%	5-helix bundle	0.127	[33, 37]
				Block 4: Q2112, R2126, Y2126. (dimerization domain)	2QDQ (*Mus musculus*)	45%	96.6	Antiparallel coiled-coil	0.220	[37]
Vinculin (KX463730)	XP_016608717 (*Spizellomyces punctatus*)	vinculin family domain (Pfam 01044)	Full length		1TR2 (*Homo sapiens*)	32%	>90%	5 amphipathic helices	1.224	[36]
Paxillin (KX463729)	NP_990315.1 (*Gallus gallus*)	LIM domains (Pfam 00412)	98–153, 157–213, 217–272, 276–335	Several conserved Cys and His residues potentially implicated in binding Zn	Partial, not modeled					[51]

Predicted 3D models of C1A scaffolding proteins are shown in Fig 5 and are detailed in S1 File. In all cases, typical characteristic structural motifs were predicted for C1A proteins (Table 2 and Fig 5) [32, 35–41]. Superimposing the predicted model on template proteins yielded very low RMSD values (Table 2), indicative of a high similarity between the superimposed atomic coordinates in C1A versus the template proteins [42]. These results above clearly demonstrate that C1A scaffolding proteins are predicted to be structurally similar to their functional metazoan counterparts.

### Genes encoding scaffolding proteins are transcribed during C1A growth even in the absence of an ECM trigger for adhesion

**Fig 6:**
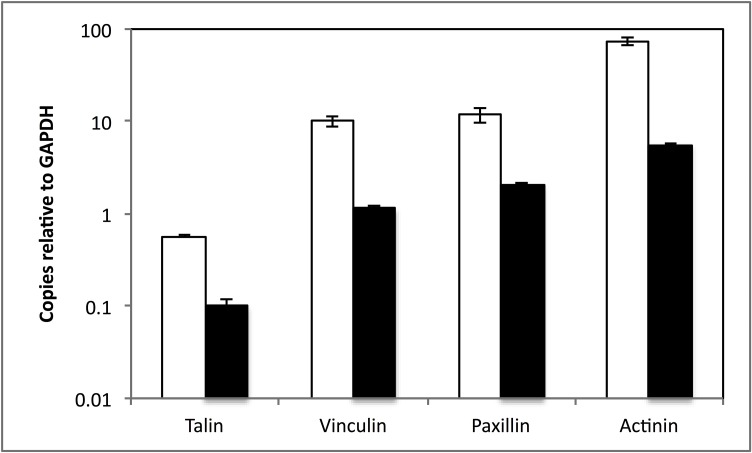
Transcriptional levels of genes encoding scaffolding proteins in the presence and absence of an extracellular matrix polysaccharide. The number of transcript copies of talin, paxillin, vinculin, and α-actinin relative to the number of transcript copies of the housekeeping gene glyceraldehyde-3-phosphate dehydrogenase (GAPDH) are shown when C1A was grown on soluble cellobiose media (☐) (i.e. in absence of an ECM) versus when grown on a MCC media (■) (i.e. in presence of an ECM). Error bars are standard deviations from two experiments (each with 2 replicates) for paxillin, vinculin, and α-actinin, and four experiments (each with two replicates) for talin. Values were significantly higher in absence of ECM for talin (5.7-fold increase, Student t-test P-value = 0.001), α-actinin (13.1-fold increase, Student t-test P-value = 0.009), vinculin (8.7-fold increase, Student t-test P-value = 0.008), and paxillin (5.7-fold increase, Student t-test P-value = 0.07).

We investigated the transcription of genes encoding the 4 scaffolding proteins in C1A cultures grown on cellobiose. C1A total mRNA contained transcripts of genes encoding all 4 scaffolding proteins in levels ranging from 0.6–73 times the level of transcription of the housekeeping gene GAPDH (Fig 6). We compared the transcriptional levels of genes encoding scaffolding proteins in the presence and absence of an insoluble extracellular polysaccharide matrix (microcrystalline cellulose). The transcriptional levels of genes encoding for all four scaffolding proteins (α-actinin, talin, paxillin, and vinculin) were significantly lower when grown on MCC, as opposed to cellobiose-grown culture (Fig 6).

### Transcripts of scaffolding proteins are up-regulated during zoosporogenesis

**Fig 7:**
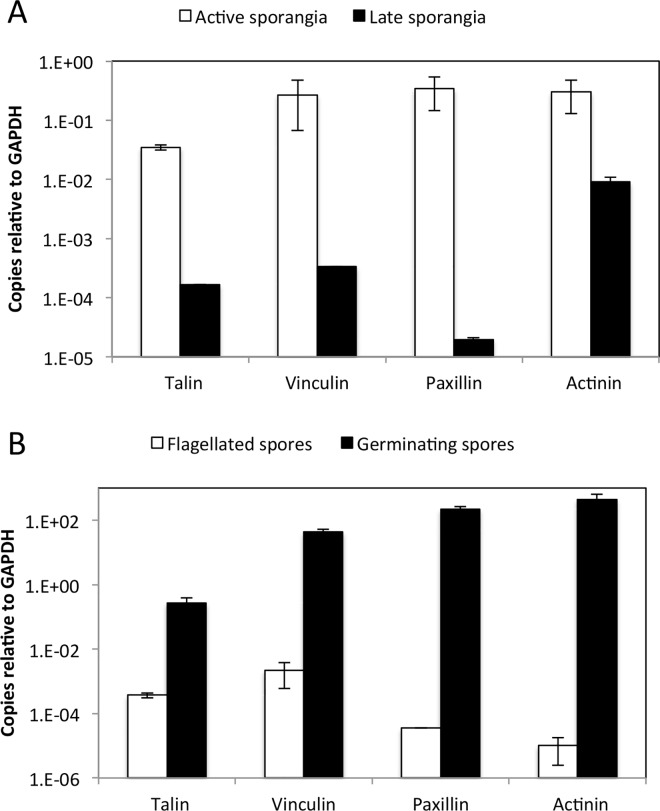
Transcriptional levels of genes encoding scaffolding proteins in various life cycle stages of C1A. The number of transcript copies of talin, paxillin, vinculin, and α-actinin relative to the number of transcript copies of the housekeeping gene glyceraldehyde-3-phosphate dehydrogenase (GAPDH) are shown for the active sporangia (☐) versus the late sporangia (■) samples (A), as well as for the flagellated spores (☐) versus germinating spores (■) samples (B). Error bars are standard deviations from two experiments (each with 2 replicates) for paxillin, vinculin, and α-actinin, and four experiments (each with two replicates) for talin. Transcriptional levels were significantly higher in active sporangia compared to late sporangia [for talin (773-fold increase, Student t-test P-value = 0.005), vinculin (807-fold increase, Student t-test P-value = 0.03), paxillin (17,463-fold increase, Student t-test P-value = 0.049), and α-actinin (33-fold increase, Student t-test P-value = 0.007)]. Likewise, transcriptional levels were significantly higher in germinating spores compared to flagellated spores [for talin (730-fold increase, Student T t-test P-value = 0.002), vinculin (19,589-fold increase, Student t-test P-value = 0.022), paxillin (6,300,742-fold increase, Student T-test p-value = 0.018), and alpha-actinin (43,513,108-fold increase, Student t-test P-value = 0.048)]. Comparing active sporangia to germinating spores, transcriptional levels were also significantly higher in germinating spores [for talin (7.8-fold increase, Student t-test P-value = 0.005), vinculin (160-fold increase, Student t-test P-value = 0.027), paxillin (647-fold increase, Student t-test P-value = 0.048), and alpha-actinin (1,494-fold increase, Student t-test P-value = 0.007)].

Next, we investigated whether the transcriptional patterns of genes encoding scaffolding proteins are dependent on C1A developmental stage. We first quantified transcription levels during active versus inactive sporogenesis. Based on microscopic examination (Table A in S1 File), and the transcriptional levels of centrin and RS3 (Fig B in S1 File) we chose day 5 as an active zoosporogenesis sample, and day 19 as an inactive late sporangia sample. We studied the transcriptional levels of α-actinin, talin, vinculin, and paxillin in such samples. Results (Fig 7A) show a significantly higher transcription level in active sporangia for α-actinin, talin, vinculin, and paxillin. The observed significant increase in the transcription levels of genes encoding scaffolding proteins during active sporogenesis implies a functional role for these proteins during the zoosporogenesis process.

### Flagellated zoospores carry scaffolding proteins mRNAs

We showed above that the genes encoding all 4 scaffolding proteins were highly expressed in the active sporangia sample. Following zoosporogenesis, zoospores are released from the active sporangia, and are motile by means of their posterior flagella. To demonstrate whether the scaffolding genes transcripts are stored with the active zoospores, we collected a swimming spores only sample [11] and used it to study the transcriptional level of α-actinin, talin, vinculin, and paxillin. We show that the zoospores total mRNA contained scaffolding genes transcripts, albeit with significantly lower transcription levels than what was observed in the active sporangia sample (Fig 7A and 7B) for α-actinin (29,119-fold decrease, Student t-test P-value = 0.007), talin (346-fold decrease, Student t-test P-value = 1E-09), vinculin (123-fold decrease, Student t-test P-value = 0.027), and paxillin (9,734-fold decrease, Student t-test P-value = 0.048). Similar results were shown before for the basal chytrid fungus *Batrachochytrium dendrobatidis*, where vinculin was found to be differentially expressed in the sporangia as opposed to the zoospore sample [25].

### Scaffolding proteins transcripts are differentially up-regulated in germinating spores as opposed to swimming spores

The presence of scaffolding proteins transcripts as part of C1A swimming spores total mRNA might suggest the utility of these proteins during encystment and germination. To test this hypothesis, we collected a germinating spores-only sample [11] and used it to examine the transcriptional level of α-actinin, talin, vinculin, and paxillin. We show that the transcription level of genes encoding all 4 scaffolding proteins was highest in germinating spores. Levels were significantly higher than in active sporangia undergoing zoosporogenesis and were also significantly higher than in swimming zoospores for all 4 genes (Fig 7). These results suggest that one of the major cellular functions of scaffolding proteins might be during and post-germination.

## Discussion

In this study, we investigated the transcriptional levels and the possible cellular functions of α-actinin, paxillin, vinculin, and talin in the anaerobic fungus strain C1A in the absence of homologues for extracellular matrix anchors (e.g. integrin). We show that α-actinin, talin, vinculin, and paxillin are actively transcribed, and their proteins are predicted to be structurally similar to their functional metazoan counterparts. Analysis of their transcriptional patterns at different stages of fungal development further demonstrated that the scaffolding proteins transcripts were detectable in mRNA from swimming zoospores, that their transcriptional levels were higher during active zoosporogenesis and highest in germinating spores.

Based on these results, we speculate on the putative functions of scaffolding FA proteins in the anaerobic gut fungi. As explained above, the absence of integrin and the presence of scaffolding proteins in basal fungal lineages (Fig 1) is notable, since integrin represents the anchor for stable contact between the extracellular matrix and the cell’s cytoskeleton. Since, in metazoan FA systems, the scaffolding proteins are interacting with integrin (directly or indirectly), it is unclear what the cellular functions of the scaffolding proteins would be in basal fungi. It is possible that other proteins (non-homologous to integrin) could replace the cellular functions of the missing ECM anchor. This has been shown before in the non-metazoan *Dictyostelium discoideum* whose genome lacks clear homologues of integrin. However, cell substratum adhesion during early development was found to occur via the membrane proteins SibA and SadA [43], both of which were shown to interact with the FA scaffolding protein talin [44]. However, when C1A was grown in the presence of the insoluble microcrystalline cellulose (to which C1A hyphae were tightly attached and excessively intertwined), genes for scaffolding protein were not differentially up-regulated (Fig 6).

Therefore, we speculate that transcription of genes encoding scaffolding proteins in C1A may reflect their involvement in non-adhesion associated functions. One possible function could be postulated based on similarities between the ciliates basal bodies and the fungal spores flagella. In ciliated eukaryotes, the scaffolding proteins paxillin and vinculin localize to ciliary adhesion complexes during ciliogenesis linking the basal bodies to the actin cytoskeleton [18]. This cellular function of scaffolding proteins did not require the presence of integrin. Interestingly, all basal fungi with sequenced genomes with clear homologues for FA scaffolding proteins have a flagellated zoospore stage. Flagella and cilia are similar in that they are both attached to a basal body [45]. It is therefore plausible that the scaffolding proteins in basal fungi could have a function in basal body formation during zoosporogenesis, and that they interact with actin much similar to what was shown in ciliated cells during ciliogenesis. Indeed, our transcriptional study showed that the genes encoding all 4 scaffolding proteins in C1A were differentially up-regulated during active zoosporogenesis compared to late sporangia. The function of scaffolding proteins during ciliogenesis [18] is similar to their function in FA [1, 8]; being interaction with the actin cytoskeleton.

In the chytrid fungi *Blastocladiella emersonii* and *Batrachochytrium dendrobatidis*, transcripts carried with the swimming zoospores encode proteins needed during encystment and germination [24, 25, 46]. We here demonstrate that C1A zoospores carry scaffolding protein transcripts during swimming, which would suggest a possible cellular function for scaffolding proteins during and/or post encystment and germination. One possibility that would require further examination is the involvement of FA scaffolding proteins in C1A hyphal tip growth. Recent studies have suggested that turgor pressure might not be the only driver for hyphal expansion [47, 48], and a role of microfilaments in hyphal tip growth has been proposed [47]. Scaffolding proteins might be functional in the hyphal tip for linking the microfilaments to the plasma membrane. Evidence to support this speculation comes from research on the chytrid fungus *Allomyces arbuscula*, where a focal adhesion-specific protease (similar to calpains in animal cells) was co-localized with microfilaments to the hyphal apical tip [49]. The preferred substrates for calpains in animal cells are focal adhesion scaffolding proteins [50]. The presence of such calpain in the fungal apical tip suggests that its substrates, the scaffolding proteins, might also be co-localized to perform a specific function during hyphal tip elongation.

Results shown here are relevant from an evolutionary standpoint. As shown before [8, 18], homologues for all focal adhesion components were identified in the genomes of representatives of Apusozoa, the Opisthokonta sister group, suggesting an ancient origin of focal adhesion that seems to predate the divergence of Opisthokonta [7]. Components of the FA machinery were independently lost during evolution [8], but the presence of scaffolding proteins homologs among all unikonts (Opisthokonts and Amoebozoa) examined (Fig 1, and [8]), prompted [18] to suggest that the scaffolding proteins might have evolved first to perform a non-adhesion-related function (e.g. flagella- or cilia-related, or hyphal tip growth in filamentous fungi) and then were later co-opted for FA. Regardless of whether scaffolding proteins assumed a non-adhesion related function following an integrin loss event [8], or were originally performing a non-adhesion-related function then were coopted for FA following an integrin gain event [18], the current study suggests that scaffolding proteins could have more diverse functionalities than originally understood.

## Supporting Information

S1 File Contains supporting text, two supplementary tables and two supplementary figures. **Table A. Microscopy results when C1A was grown in cellobiose (or MCC) media over a period of 19 days. Table B. Blastp results in other Neocallimastigomycota transcriptomes (from [30]). Fig A. C1A predicted paxillin Pfam domain organization.** (A), and pairwise sequence alignment of to paxillin from *Gallus gallus* (NP_990315).**Fig B. Transcriptional levels of genes encoding RS3 and centrin in C1A.** The number of transcript copies of RS3 (☐) and centrin (ѵ) relative to the number of transcript copies of the housekeeping gene glyceraldehyde-3-phosphate dehydrogenase (GAPDH) were followed over a period of 19 days. Error bars are standard deviations from two experiments (each with 2 replicates).
(DOCX)
