# Supplementary material for: Insights into the Utility of the Focal Adhesion Scaffolding Proteins in the Anaerobic Fungus Orpinomyces sp. C1A
Source: PLoS One. 2016 Sep 29;11(9):e0163553. doi: 10.1371/journal.pone.0163553 (PMC5042518; doi:10.1371/journal.pone.0163553)
Supplement: S1 File — Table A. Microscopy results when C1A was grown in cellobiose (or MCC) media over a period of 19 days. Table B. Blastp results in other Neocallimastigomycota transcriptomes (from [30]). Fig A. C1A predicted paxillin Pfam domain organization. (A), and pairwise sequence alignment of to paxillin from Gallus gallus (NP_990315).Fig B. Transcriptional levels of genes encoding RS3 and centrin in C1A. The number of transcript copies of RS3 (☐) and centrin (ѵ) relative to the number of transcript copies of the housekeeping gene glyceraldehyde-3-phosphate dehydrogenase (GAPDH) were followed over a period of 19 days. Error bars are standard deviations from two experiments (each with 2 replicates). (DOCX) [file pone.0163553.s001.docx]

**Supplementary material for:**

**Insights into the utility of the focal adhesion scaffolding proteins in the anaerobic fungus *Orpinomyces* sp. C1A.**

Shelby Calkins^1^ and Noha Youssef^1*^

^1^Department of Microbiology and Molecular Genetics, Oklahoma State University, Stillwater, OK 74078 USA

*Corresponding author. Mailing address 1110 S Innovation way, Stillwater, OK 74074. Phone: (405) 744-1192, Fax: (405) 744-1112. email: [noha@okstate.edu](mailto:Mostafa@okstate.edu)

Keywords: Anaerobic fungi, focal adhesion, scaffolding proteins, talin, vinculin, paxillin, α-actinin, flagellar assembly, zoosporogenesis, hyphal tip growth.

**I. S Text:**

**Detailed analysis of C1A predicted scaffolding proteins.**

1. Alpha-actinin: Comparison against the Pfam database showed that C1A predicted alpha-actinin harbored one actin binding domain comprised of 2 Calponin homology (CH) (Pfam 00307) domains, one spectrin repeat (Pfam 00435), and one Ca^2+^ insensitive EF hand (EF) domain (Pfam08726), a domain organization consistent with alpha-actinin from metazoan origin [1] (Figure 2A-I). Due to the partial coverage of C1A genome, the first of the two CH domains was only partial while the second spanned residues 66-169. The presence of two CH domains within the actin-binding domain was shown before to be essential for binding actin [2]. Downstream of the actin-binding domain, the spectrin repeat domain spanned residues 316-408. A closer look at the primary sequence of C1A spectrin repeat in comparison to Pfam00435 HMM profile identified the presence of the aromatic residues Y317 and Y397 and the residue L428, all of which are characteristic of the spectrin repeat. Finally, the Ca^2+^ insensitive EF hand (EF) domain identified downstream of the spectrin repeat spanned residues 505-564. Secondary structure alignments and predicted 3D structure models for each of the alpha-actinin domains are shown in Figure 2A. Due to the partial nature of the first CH domain in C1A alpha-actinin, only the second CH domain could be modeled with confidence. We utilized Phyre2 to predict the secondary structure as well as the 3D model of C1A alpha-actinin in comparison to the chicken gizzard smooth muscle alpha-actinin (PDB ID: 1SJJ). C1A CH domain was 60% similar to the corresponding 1SJJ domain and was modeled with 100% confidence. The predicted model of C1A CH domain displayed the typical structural motif of 4 alpha helices [3], and when superimposed with 1SJJ, it aligned with an RMS value of 0.941 (Figure 2A-II). C1A spectrin repeat was 27% similar to the corresponding 1SJJ domain and was modeled with 98.93% confidence. The proposed model of C1A spectrin domain maintained the typical triple-helical coiled-coil motif [4], and superposition with 1SJJ gave an RMS of 0.956 (Figure 2A-III). Finally, C1A EF domain was 32% similar to the corresponding 1SJJ domain and was modeled with 99.60% confidence. The predicted model of C1A’s alpha-actinin Ca^2+^ insensitive EF hand domain maintained the helix-loop-helix motif typical of EF hands [5]. Superposition of C1A’s alpha-actinin EF domain with 1SJJ yields a RMS value of 1.119 (Figure 2A-IV).

2. Talin: The Pfam analysis revealed that C1A talin contained 4 domains: a middle domain (Pfam 09141), 2 vinculin binding site domains (VBS1 and VBS2) (Pfam 08913), and an I/LWEQ domain (Pfam 01608) (Figure 2B-I). Talin middle domain spanned residues 100-262. C1A middle domain of talin was modeled with 100% confidence and 30% sequence identity to 1SJ7 (Figure 2B-II). The predicted model showed the characteristic bundle comprised of 5 alpha helices [6], and the superimposed structures of the middle domain and 1SJ7 (residues 491-652) yielded an RMS of 0.116 (Figure 2B-II). Vinculin binding site domain 1 (VBS1) spanned residues 838-961 and was modeled with 100% confidence and 30% sequence identity to 2L10 (Figure 2B-III), while vinculin binding site 2 (VBS2) spanned residues 1455-1578 and was modeled with 97% confidence and 21% sequence identity to 2KVP (Figure 2B-IV). Both VBS domain predictions yielded models that were composed of 4 alpha helices characteristic of vinculin binding sites [7]. Superimposing C1A vinculin binding sites with their templates tertiary structures gave an RMS of 0.902 (VBS1), and 0.653 (VBS2). A closer look at the primary sequence of C1A talin I/LWEQ domain spanning residues 1991-2139 revealed the highly conserved 4-block structure characteristic of this domain [8]. Block 1 (residues 1952-1977) showed several conserved branched chain residues and Q1975, block 2 (residues 1996-2018) showed the conserved W1996 and several non-polar residues, block 3 (residues 2029-2053) showed the conserved E2029, Q2044, and K2052, and block 4 (residues 2109-2128) showed the conserved residues Q2112, R2126, and Y2126. Blocks1-3 of C1A I/LWEQ domain of talin were modeled with 100% confidence and 53% sequence identity to 2JSW (Figure 2B-V), while block 4 was modeled with 96.6% confidence and 45% identity to the dimerization domain 2QDQ. The predicted model showed the characteristic 5-helix bundle [9]. Superimposing C1A I/LWEQ domain on 2JSW, and 2QDQ gave an RMS value of 0.127, and 0.220, respectively (Figure 2B-V).

3. Vinculin: Pfam search revealed that C1A vinculin only had 1 domain, the vinculin family domain (Pfam 01044) (Figure 2C-I). The available C1A vinculin sequence only spans the C-terminal region of the protein, where it aligned with residues 804-1061 of the human full-length vinculin (PDB: 1TR2) (Figure 2C-II). C1A vinculin domain was modeled with x% confidence and 32% sequence identity to 1TR2 (Figure 2C-II). The predicted model showed 5 amphipathic helices characteristic of the vinculin tail [10]. C1A vinculin superimposed with 1TR2 tail with an RMS value of 1.224 (Figure 2C-II).

4. Paxillin: The C1A predicted paxillin protein was only a partial sequence. Blastp comparison against the nr database identified the paxillin from *Gallus gallus* (Genbank accession number NP_990315.1) as its first hit with 57% sequence similarity (alignment to NP_990315.1 is shown in Figure 1). Comparison against the Pfam database identified 4 LIM domain (Pfam 00412) spanning residues (98-153), (157-213), (217-272), (276-335). A closer look at the primary sequence of each LIM domain and its comparison to the Pfam HMM profile identified several conserved histidine and cysteine residues that are potentially implicated in binding Zn (Figure S2). Since the C1A predicted paxillin available sequence did not span the paxillin domain itself, it was not possible to perform any secondary or tertiary structure predictions using the available paxillin proteins in the PDB database.

**II. Supplementary tables:**

**Table A.** Microscopy results when C1A was grown in cellobiose (or MCC) media over a period of 19 days.

|  | Spores | | Sporangia | |
| --- | --- | --- | --- | --- |
| Sample | Swimming (flagellated) | Resting (not flagellated) | Swimming spores inside | Without swimming spores inside |
| Day 1 | ++ | +++ | - | + |
| Day 5 | +++ | ++ | +++ | +++ |
| Day 15 | + | ++ | ++ | ++ |
| Day 19 | + | ++ | + | +++ |

Scale: (-) none observed, (+) very few observed, (++) some observed, (+++) many observed.

**Table B.** Blastp results in other Neocallimastigomycota transcriptomes (from [11]).

| FA component | | Hits in^a^ | | |
| --- | --- | --- | --- | --- |
|  |  | *Anaeromyces* | *Piromyces* | *Neocallimastix* |
| Anchor | Integrin | N | N | N |
| Signaling kinases | FAK | N | N | N |
|  | Src | N | N | N |
| Scaffolding proteins | Talin | Y (g.6332) | Y (g.13026) | Y (g.12367) |
|  | Vinculin | Y (g.7072) | Y (g.15720) | Y (g.8754) |
|  | Paxillin | Y (g.3771) | Y (g.3279) | Y (g.6669) |
|  | α-actinin | Y (g.3928) | Y (g.3512) | Y (g.2424) |
| IPP complex | PINCH | Y (g.5606) | Y (g.16322) | Y (g.13985) |
|  | Parvin | Y (g.11341) | Y (g.1163) | Y (g.12919) |
|  | ILK | Y (g.9193) | Y (g.2454) | Y (g.9692) |

a: Criteria used for Blastp were a minimum of 50% alignment length and > 30% similarity. N: no homologues identified, Y: a homologue was identified.

**III. Supplementary Figures.**

**Figure A**. C1A predicted paxillin Pfam domain organization (A), and pairwise sequence alignment of to paxillin from *Gallus gallus* (NP_990315).

B


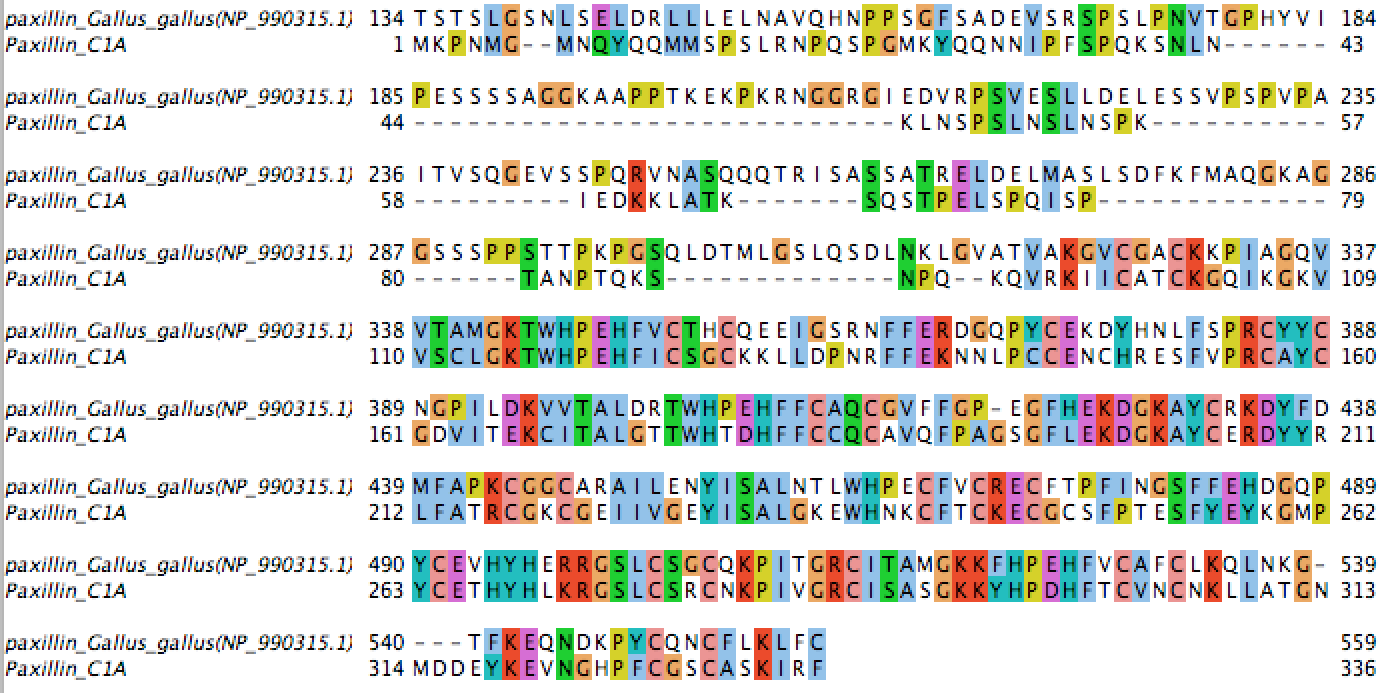

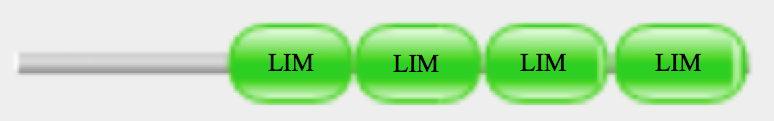


A

**Figure B**. **Transcriptional levels of genes encoding RS3 and centrin in C1A.** The number of transcript copies of RS3 (☐) and centrin (◼) relative to the number of transcript copies of the housekeeping gene glyceraldehyde-3-phosphate dehydrogenase (GAPDH) were followed over a period of 19 days. Error bars are standard deviations from two experiments (each with 2 replicates).

**References**

1. Sjöblom B, Salmazo A, Djinović-Carugo K. α-Actinin structure and regulation. Cell Mol Life Sci. 2008;65(17):2688-701. doi: 10.1007/s00018-008-8080-8.

2. Stradal T, Kranewitter W, Winder SJ, Gimona M. CH domains revisited. FEBS letters. 1998;431(2):134-7. Epub 1998/08/26. PubMed PMID: 9708889.

3. Korenbaum E, Rivero F. Calponin homology domains at a glance. Journal of cell science. 2002;115(18):3543-5. doi: 10.1242/jcs.00003.

4. Yan Y, Winograd E, Viel A, Cronin T, Harrison SC, Branton D. Crystal structure of the repetitive segments of spectrin. Science (New York, NY). 1993;262(5142):2027-30. Epub 1993/12/24. PubMed PMID: 8266097.

5. Atkinson RA, Joseph C, Kelly G, Muskett FW, Frenkiel TA, Nietlispach D, et al. Ca2+-independent binding of an EF-hand domain to a novel motif in the alpha-actinin-titin complex. Nature structural biology. 2001;8(10):853-7. Epub 2001/09/27. doi: 10.1038/nsb1001-853. PubMed PMID: 11573089.

6. Papagrigoriou E, Gingras AR, Barsukov IL, Bate N, Fillingham IJ, Patel B, et al. Activation of a vinculin-binding site in the talin rod involves rearrangement of a five-helix bundle. EMBO J. 2004;23(15):2942-51. Epub 2004/07/24. doi: 10.1038/sj.emboj.7600285. PubMed PMID: 15272303; PubMed Central PMCID: PMCPMC514914.

7. Gingras AR, Vogel KP, Steinhoff HJ, Ziegler WH, Patel B, Emsley J, et al. Structural and dynamic characterization of a vinculin binding site in the talin rod. Biochemistry. 2006;45(6):1805-17. Epub 2006/02/08. doi: 10.1021/bi052136l. PubMed PMID: 16460027.

8. McCann RO, Craig SW. The I/LWEQ module: a conserved sequence that signifies F-actin binding in functionally diverse proteins from yeast to mammals. Proc Natl Acad Sci USA. 1997;94(11):5679-84. Epub 1997/05/27. PubMed PMID: 9159132; PubMed Central PMCID: PMCPMC20838.

9. Gingras AR, Bate N, Goult BT, Hazelwood L, Canestrelli I, Grossmann JG, et al. The structure of the C-terminal actin-binding domain of talin. EMBO J. 2008;27(2):458-69. Epub 2007/12/25. doi: 10.1038/sj.emboj.7601965. PubMed PMID: 18157087; PubMed Central PMCID: PMCPMC2168396.

10. Bakolitsa C, de Pereda JM, Bagshaw CR, Critchley DR, Liddington RC. Crystal structure of the vinculin tail suggests a pathway for activation. Cell. 1999;99(6):603-13. doi: <http://dx.doi.org/10.1016/S0092-8674(00)81549-4>.

11. Solomon KV, Haitjema CH, Henske JK, Gilmore SP, Borges-Rivera D, Lipzen A, et al. Early-branching gut fungi possess a large, comprehensive array of biomass-degrading enzymes. Science (New York, NY). 2016;351(6278):1192-5. doi: 10.1126/science.aad1431.
